# Direct observation of spinodal decomposition phenomena in InAlN alloys during *in-situ* STEM heating

**DOI:** 10.1038/srep44390

**Published:** 2017-03-14

**Authors:** J. Palisaitis, C.-L. Hsiao, L. Hultman, J. Birch, P. O. Å. Persson

**Affiliations:** 1 Thin Film Physics Division, Department of Physics, Chemistry and Biology (IFM), Linköping University, SE-581 83 Linköping, Sweden

## Abstract

The spinodal decomposition and thermal stability of thin In_0.72_Al_0.28_N layers and In_0.72_Al_0.28_N/AlN superlattices with AlN(0001) templates on Al_2_O_3_(0001) substrates was investigated by *in-situ* heating up to 900 °C. The thermally activated structural and chemical evolution was investigated in both plan-view and cross-sectional geometries by scanning transmission electron microscopy in combination with valence electron energy loss spectroscopy. The plan-view observations demonstrate evidence for spinodal decomposition of metastable In_0.72_Al_0.28_N after heating at 600 °C for 1 h. During heating compositional modulations in the range of 2–3 nm-size domains are formed, which coarsen with applied thermal budgets. Cross-sectional observations reveal that spinodal decomposition begin at interfaces and column boundaries, indicating that the spinodal decomposition has a surface-directed component.

III-nitride semiconductors attract attention for applications in optoelectronic devices owing to a direct bandgap, tenable from 0.6 eV to 6.2 eV^1^. However, alloys in the wurtzite-structure (Al, Ga, In)N system prove challenging to grow throughout the full compositional range. This is due to a wide miscibility gap for the pseudo-binaries^2^. The thermodynamic stability of the InGaN system was investigated by Ho and Stringfellow^3^, who found a pronounced miscibility gap at normal growth temperatures, owing to a large difference in interatomic spacing between the binary constituents InN and GaN. In parallel, observations by electron microscopy revealed In-rich dot-like features in InGaN quantum wells^4^,^5^,^6^, which are thought to result from spinodal decomposition. Similarly, for InAlN there exists a large miscibility gap though AlGaN is not expected to decompose^2^,^7^,^8^. Spinodal decomposition is a common and technically beneficial process^9^ for a number of metals and ceramics^10^,^11^ such as TiAlN.

The realization of InAlN alloys has been demonstrated by a number of growth techniques which includes magnetron sputter epitaxy (MSE)^12^, metalorganic vapor deposition^13^ and molecular beam epitaxy^14^. If growth performed at temperatures by near or at thermal equilibrium, the material was suggested to undergo segregation through spinodal decomposition already during growth to Al-rich and In-rich InAlN domains. MSE owns the advantage of permitting growth of epitaxial InAlN films at temperatures far below thermal equilibrium. This allows to cover the whole InN-AlN compositional range, including compositions inside the miscibility gap, without the onset of phase separation^15^,^16^.

Through access to the range of compositions the thermal development of this material allows for a comprehensive investigation. Consequently, the present manuscript presents direct experimental evidence for spinodal decomposition in the InAlN system, as revealed by *in-situ* heating in a scanning transmission electron microscope (STEM). The spinodal decomposition was followed during heating of as-grown In_0.72_Al_0.28_N solid solution layers and In_0.72_Al_0.28_N/AlN superlattices, where the composition of the alloy was targeted deep inside the miscibility gap. Both Al and In segregate in In_0.72_Al_0.28_N layers into compositionally separated nanometre-size domains. Additional heating coarsens the domain size with increasing compositional differences from the original layer.

## Results and Discussion

**Figure 1:**
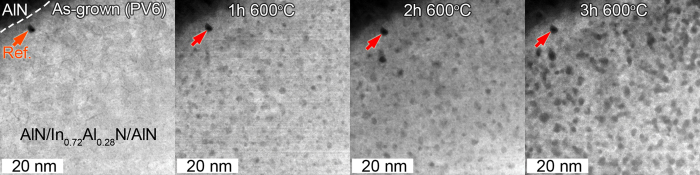
Plan-view STEM-HAADF images of single In_0.72_Al_0.28_N layer, sandwiched between two AlN layers (AlN/In_0.72_Al_0.28_N/AlN), displaying spinodal decomposition and domain coarsening during *in-situ* heating.

A time series of plan-view STEM-HAADF images displaying the evolution of single 6 nm thick In_0.72_Al_0.28_N layer (PV6), sandwiched between AlN layers, during *in-situ* heating is shown Fig. 1.

The as-grown PV6 sample exhibits a homogeneous bright contrast, such that the as-grown In_0.72_Al_0.28_N layer has no indications of phase separation. During *in-situ* heating, the initially homogeneous In_0.72_Al_0.28_N layer develop mass-contrast undulations caused by the segregation of subnanometer scale In-rich (bright) and In-deficient (dark) domains. These are visible already after 1 h at 600 °C and coarsen with time such that distinctly 2–3 nm-size dark and bright domains are visible after 3 h at 600 °C.

**Figure 2:**
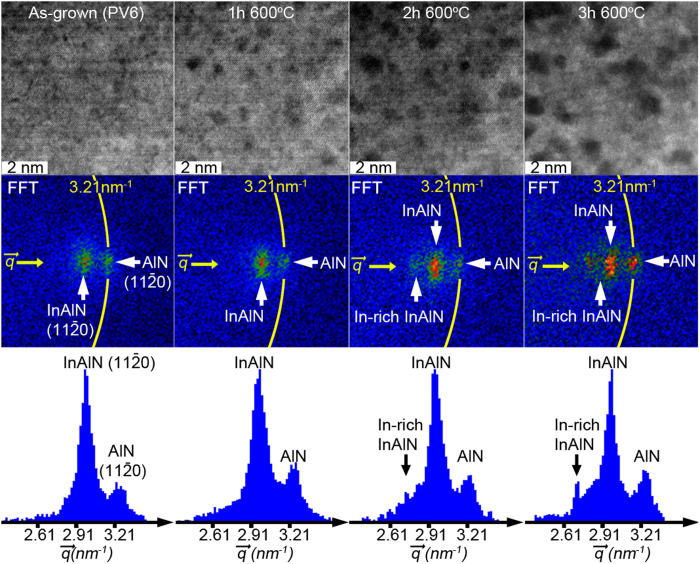
Plan-view HRSTEM-HAADF images of single In_0.72_Al_0.28_N layer, sandwiched between two AlN layers (AlN/In_0.72_Al_0.28_N/AlN), displaying the structural coarsening during *in-situ* heating are shown in the top-row. FFTs’ selections of reciprocal space displaying [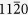] reflection are shown in the middle-row. Radially integrated FFTs’ selection intensity distributions are shown in bottom-row.

In order to assess the structural coarsening of the In_0.72_Al_0.28_N layer, a series of HRSTEM-HAADF images together with corresponding [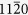] reflection intensities (extracted from Fast Fourier Transforms (FFT)) were obtained during *in-situ* heating and shown in Fig. 2.

The as-grown and 1 h heated sample FFTs reveal only the In_0.72_Al_0.28_N and AlN lattice peaks, although noticeable decomposition has been initiated in the 1 h case. However, after heating for 2 h at 600 °C indications for a shoulder (left side of the peak) development of the In_0.72_Al_0.28_N peak are visible. Additionally, a minor peak with decreased reciprocal spacing becomes visible and indicates the appearance of domains with increased In composition. After 3 h at 600 °C the new reciprocal lattice peak is well established and is at his point separated by a larger distance from the reference AlN peak (compared to 2 h).

Metastable In_0.72_Al_0.28_N layer, confined within AlN diffusion barriers, segregation into compositionally diverging nanoscale domains during heating as confirmed by the HRSTEM-HAADF and FFT (Figs 1 and 2). These observations validate that In_0.72_Al_0.28_N segregation can only originate from and is a fingerprint of spinodal decomposition predicted by phase diagrams^2^,^3^.

In order to corroborate our plan-view observation and assess the effects of In_0.72_Al_0.28_N/AlN interfaces and In_0.72_Al_0.28_N layer thicknesses on the spinodal decomposition phenomena – cross-sections of different period In_0.72_Al_0.28_N/AlN supperlattices were heated *in-situ* in the STEM.

**Figure 3:**
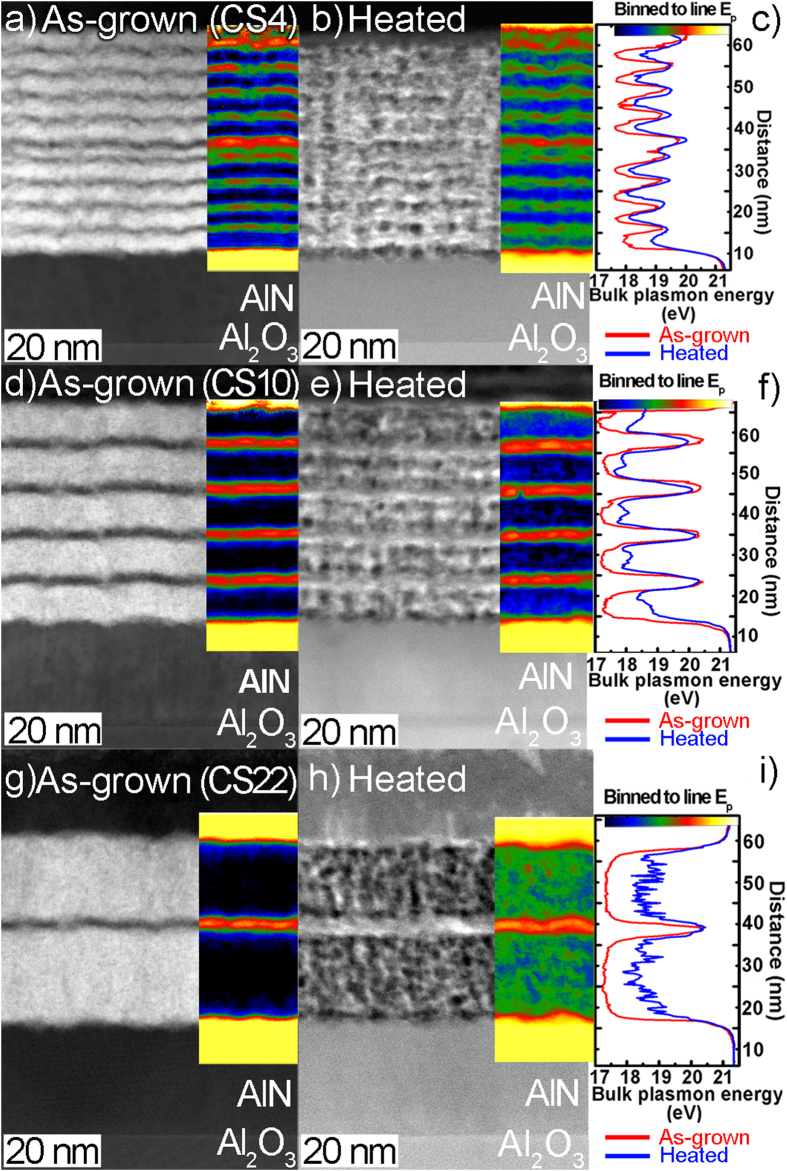
Cross-sectional STEM-HAADF images of (**a,d** and **g**) as-grown and (**b,e** and h) heated In_0.72_Al_0.28_N/AlN superlattices with different periods. The color coded overlay shows the bulk plasmon peak energy (E_p_) maps of investigated structures. Projected bulk plasmon peak energy profiles across the investigated structures are displayed in (**c,f** and **3**).

An overview STEM-HAADF images of the as-grown superlattice films (Fig. 3a,d and h) revealing the In_0.72_Al_0.28_N layers (bright contrast) separated by the AlN diffusion barrier layers (darker contrast) as well as parts of the AlN cap layer, the high temperature AlN template layer, and the Al_2_O_3_ substrate.

The 10, 5, and 2 periods of the CS4, CS10, and CS22 are shown in Fig. 3a,d and g, respectively. The as-grown samples exhibit a columnar structure and moderately smooth interfaces of the In_0.72_Al_0.28_N/AlN layers, which are induced by the uneven surface of underlying HT-AlN template layer^15^. No indications of phase separation were observed in these as-grown In_0.72_Al_0.28_N layers.

The corresponding STEM-HAADF images of the heated samples are shown in Fig. 3b,e and h. Contrast for STEM-HAADF images acquired from heated samples was enhanced in order to highlight the microstructural changes in the In_0.72_Al_0.28_N layers. The original contrast images are presented in Fig. S1. The overall superlattice structure remains intact in all samples; however, the In_0.72_Al_0.28_N layers display an irregular and varying structure in contrast to the as-grown homogeneous layers, consistent with compositional fluctuations.

The compositional state of the as-grown and heat-treated samples was monitored by STEM-VEELS SI and bulk plasmon peak energy (E_p_) mapping, which enables compositional information of In_x_Al_1−x_N^17^,^18^. The corresponding E_p_ maps are shown as overlays of the mapped locations in the STEM-HAADF images of Fig. 3a,b,d,e,g and h. The yellow color in the E_p_ maps is equivalent of AlN while black corresponds to In_0.72_Al_0.28_N. For a lateral view of the E_p_ variations across the superlattices, the projected line profiles are shown in Fig. 3c,f and i.

According to experimentally obtained E_p_ dependences on alloys composition^19^, the as-grown In_x_Al_1−x_N layer with x = 72% should display the E_p_ ~17.1 eV. This E_p_ values, for InAlN layers, were obtained in both CS10 and CS22 samples, as can be seen from the line profiles (Fig. 3f,i). However, the E_p_ for as-grown InAlN layers reach an average value of ~17.6 eV in CS4 sample indicated lower In content (x = 0.63) in the layer (Fig. 3c) although the identical growth conditions were applied as for all samples (see Methods section). Furthermore, the original E_p_ value for AlN is 21.2 eV as observed in e.g. AlN buffer layers (Fig. 3c,f and i). However, the AlN barrier layers in as-grown CS4 exhibit an average E_p_ of ~19.4 eV (x = 0.31) and E_p_ ~20.4 eV (x = 0.14) for both CS10 and CS22.

The reduced E_p_ value of the AlN barrier layers throughout the superlattices are suggested to come as a consequence of the columnar nature and waviness of the interfaces, as well as delocalization of the bulk plasmon and effects from the convergent probe^20^. The absence of In in AlN barriers were further collaborated with EDX mapping (not shown).

Underestimation of E_p_ in AlN and overestimation of E_p_ In_0.72_Al_0.28_N layer are the most pronounced in CS4. While due to slightly better interfacial quality, increased In_0.72_Al_0.28_N layer thicknesses and resultant smaller overlap inside the layers of the bulk plasmons’ this effect is less pronounced in CS10 and CS22.

The as-grown CS4 is shown in Fig. 3a. Due to the columnar structure and interfacial waviness, all layers are better separated inside the columns in contrast to the columnar boundaries. The heated CS4 sample sustains the original periodicity, but the superlattice structure appears significantly distorted, with spot-like domains of the order of 2–3 nm (Fig. 3b).

The E_p_ map of the as-grown and heated CS4 sample confirms the preserved periodicity during heating of the superlattice (Fig. 3a,b). It further suggests a small lateral variation in concentration, as a result of spinodal decomposition inside the layers. The projected line profile in Fig. 3c (blue line) shows a shift of the E_p_ value to ~18.1 eV (from original ~17.6 eV) inside the layers, which indicates a loss of In from the InAlN layers. It was previously shown that In can desorb through the surfaces of a thin TEM foil during high-temperature heating^21^, which the present cross-section sample symmetry would allow. Finally, the top and bottom In_0.72_ Al_0.28_N layers are seen to have the same average E_p_ as the other layers in the as-grown sample, while for the heated sample, these layers loose more In compared to the adjacent. This is suggested to occur as a consequence of the more stable interfaces of the AlN buffer and cap layers.

The as-grown CS10 is shown in Fig. 3d. As with CS4, the layer structure is columnar and wavy. The heated CS10 sample exhibits doubly-modulated layers in the original In_0.72_Al_0.28_N layers (Fig. 3e). The E_p_ maps of the as-grown and heated CS10 samples are shown as overlays on the STEM-HAADF images in Fig. 3d,e, respectively, along with the projected line profile (Fig. 3f). The plasmon spectrum of the heated CS10 (Fig. 3e) clearly identifies the additional layering inside the 10 nm thick In_0.72_Al_0.28_N layers, as a compositional split of the layer, which is shown by the projected E_p_ variations across the superlattice (Fig. 3e blue line). The original E_p_ at ~17.1 eV within the In_0.72_Al_0.28_N layer experiences a shift to ~17.9 eV (x = 0.58) and ~18.2 eV (x = 0.52) in the appearing internal spacer layer. The presence of AlN interlayers was also confirmed with STEM-HAADF image intensity analysis (see Fig. S2). The E_p_ of the AlN barrier layers together with barrier width remains similar. As with CS4 a general shift of E_p_ is observed as a result of In loss. Also in this sample, E_p_ is found to exhibit higher energy gain at the top and bottom interface of the superlattices, explained as above to occur through differences in the structural quality at these interfaces. See, e.g., the particularly dark bottom interface.

Finally, the as-grown CS22 is shown in Fig. 3g. The heated CS22 sample reveals a preserved 24 nm period structure, however, it is apparent that the original In_0.72_Al_0.28_N layer has been segregated into a pronounced nanoscale pattern of 2–3 nm large domains (Fig. 3h). Also, darker layers are observed along the interfaces, and bright thin lines are seen to protrude from the top layer and into the AlN cap. The E_p_ maps and projected profiles of the as-grown and heated CS22 samples are shown in Fig. 3g–i, respectively. The as-grown layers exhibit a uniform composition as seen by the slowly varying E_p_ (~17.1 eV) corresponding to an average composition of In_0.72_Al_0.28_N. The AlN barrier layers are also clearly defined with an average E_p_ at ~20.4 eV with locally higher values. The heatedline profile exhibit an average E_p_ at ~18.5 eV due to loss of In (Fig. 3i). It can be seen that the segregated structure is reflected in the E_p_ map, clearly showing that compositional variations are the reason for the observed structure and not from thickness variations.

**Figure 4:**
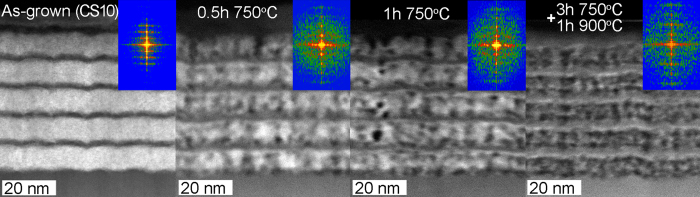
Cross-sectional STEM-HAADF images of In_0.72_Al_0.28_N/AlN (10 nm/2 nm) superlattice (CS10) showing the evolution during *in-situ* heating. The inset shows FFT from investigated structures.

**Figure 5:**
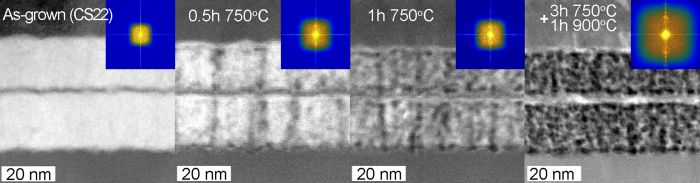
Cross-sectional STEM-HAADF images of In_0.72_Al_0.28_N/AlN (22 nm/2 nm) superlattice (CS22) showing the evolution during *in-situ* heating. The inset shows FFT from investigated structures.

The images in Fig. 3 were obtained from the as-grown state as well as after a full heating cycle as described in experimental details. To follow the structural developments during heating, a series of STEM-HAADF images were obtained from CS10 and CS22 at and shown in Figs 4 and 5, respectively.

The structural evolution of the CS10 sample is shown in Fig. 4 together with corresponding FFT patterns obtained from the superlattice structure and acquired during *in-situ* heating.

As can be seen by the STEM-HAADF contrast variations during *in-situ* heating, initially the In_0.72_Al_0.28_N layers exhibits segregation by the formation of In-rich (bright dots) and In-deficient (darker) domains already after 0.5 h at 750 °C there exists a clear onset of spinodal decomposition, judging from the apparent mass-contrast alone. Also, the associated FFT (inset) indicates a reduced period along the growth direction. There also appears to be a distinct and advanced onset of spinodal decomposition at the bottom In_0.72_Al_0.28_N surfaces, such that a darker line is formed. The original In_0.72_Al_0.28_N layers split formation into compositionally doubly-modulated can be seen already after 1 h at 750 °C and it becomes more pronounced with time and is completely present after full heating cycle. The apparent change in the layer periodicity is well resolved in the associated FFT patterns shown in the insets of Fig. 4.

Upon spinodal decomposition the domains formed in the cross-section are of the order of 2–3 nm at 700 °C, and they coarsen with time and temperature. This is in agreement with plan-view single-layer results presented in Figs 1 and 2. These results prove that the onset of spinodal decomposition preferably occurs at interfaces and at column boundaries. It is well established that boundary layer(s) or free surfaces stimulates the presence of one phase preferentially formed at that boundary/free surface of the system undergoing spinodal decomposition^22^.

With the reduced degrees of freedom in these thin layers the elemental diffusion paths are limited, which results in spinodal decomposition with a wave vector directed normal to the interface/boundary, also known as surface directed spinodal decomposition (SDSD)^23^. SDSD is suggested to cause a compositionally doubly-modulated structure, with its undulation perpendicular to the interfaces, within 10 nm thick In_0.72_Al_0.28_N layers (CS10).

A similar evolution may be observed in the 22 nm thickness In_0.72_Al_0.28_N layers (CS22) during *in-situ* heating as shown in Fig. 5.

The segregation is only present at domain boundaries and at interfaces at 0.5 h at 750 °C, while the interior of the In_0.72_Al_0.28_N layer appears intact to a large extent. Hence, the segregation appears to be slower in these layers, but promoted again by grain boundary and interface diffusion where such are present. Heated layers exhibit a uniform compositional line along the interface, brought about by SDSD, but exhibit a random structure inside the thick layer, which is to be expected from conventional spinodal decomposition in larger volumes, where surfaces are less dominating and contrasting to CS10 sample.

In the cross-sectional superlattices, initial spinodal decomposition proceeds by forming bright and grey areas in the In_0.72_Al_0.28_N layers, corresponding to regions with elevated and reduced In content. The observation is in agreement with the results from the plan-view sample (PV6), presented in Figs 1 and 2. One can also observe the formation of local dark spots, during the initial stages particularly in locations, where the segregation is initiated, i.e. at boundaries and interfaces. The dark spots are found to exhibit a low E_p_ (Fig. 3h,i), such that they are In rich. On the other hand, these appear dark in the STEM-HAADF images, suggesting that they identify regions of less mass (density) attributed to the loss of In through the TEM-sample surfaces. Hence, eventually there is no driving force for further decomposition after a prolonged heating cycle. The reduced In content domains are also expected to be stable at the present temperatures, as the thermal stability of InAlN is increased with increasing Al content^21^. Thus, the final product is a porous skeletal structure which does not evolve further, as seen in the STEM-HAADF images of the fully heated samples.

## Conclusions

The microstructure development of metastable single In_0.72_Al_0.28_N layers and In_0.72_Al_0.28_N/AlN superlattices was studied by means of *in-situ* STEM heating in a temperature range from 600 °C to 900 °C. A plan-view geometry investigation revealed that In_0.72_Al_0.28_N layer separates into In-enriched and In-depleted nanoscale domains, with increasing time and temperature, according to spinodal decomposition. It was further shown that for cross-sectional samples with increasing In_0.72_Al_0.28_N layer thicknesses in the In_0.72_Al_0.28_N/AlN superlattices, the spinodal decomposition is initiated at interfaces and boundaries, which proves that the decomposition is surface directed. For 10 nm thick In_0.72_Al_0.28_N layers in superlattices, the surface directed spinodal decomposition becomes dominant and cause each single-phase layer to split into compositionally doubly-modulated layer, while for 22 nm thick In_0.72_Al_0.28_N layers the spinodal structure is more isotropic.

## Methods

The samples comprising single layers In_0.72_Al_0.28_N and In_0.72_Al_0.28_N/AlN superlattices were grown by ultra-high vacuum MSE. High-purity 75 mm-diameter aluminum (99.999%) and 50 mm-diameter indium (99.999%) targets were used to either co-sputter ternary InAlN or sputter binary AlN layers under pure nitrogen ambient. The Al and In magnetron powers were kept constant at 50 and 10 W, respectively, in order to grow Al_0.28_In_0.72_N alloy. The growth of In_0.72_Al_0.28_N/AlN superlattices with designed layer thicknesses were achieved while manipulating shutters’ (installed in front of targets) open time periods. A detailed description of the growth conditions used for similar structures can be found elsewhere^15^.

For plan-view (PV) analysis, a single In_0.72_Al_0.28_N layer of 6 nm thickness was grown at room temperature (RT) on high-temperature (HT) 1000 °C AlN(0001) template layer on Al_2_O_3_(0001) substrates, and capped by a RT-AlN layer. Henceforth, the sample is referred according to its observation direction (plan-view) and In_0.72_Al_0.28_N layer thickness (6 nm) as PV6. For cross-sectional (CS) analysis, three In_0.72_Al_0.28_N/AlN superlattices containing 10 periods of 6 nm (4 nm/2 nm), 5 periods of 12 nm (10 nm/2 nm), and 2 periods of 24 nm (22 nm/2 nm), respectively, were grown at RT on HT-AlN(0001) template layer on Al_2_O_3_(0001), and capped by a RT-AlN layer. AlN layers are acting as thermally stable diffusion barrier in the grown structures^24^.

**Figure 6:**
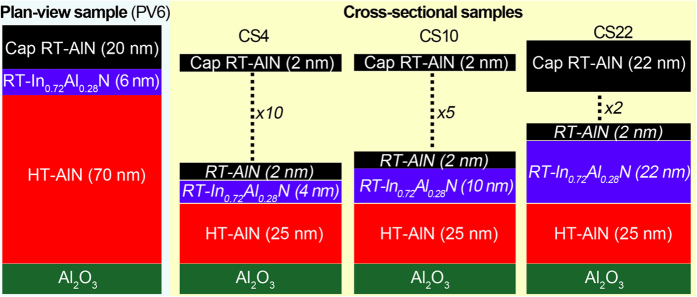
Structure schematic illustration for single-layer In_0.72_Al_0.28_N and In_0.72_Al_0.28_N/AlN superlattice samples used for plan-view and cross-sectional investigation, respectively.

The samples are referred as CS4, CS10 and CS22. The CS4 and CS10 samples have a total superlattice thickness of 60 nm, while the CS22 sample has a thicker AlN cap for a total thickness of 70 nm. The schematic structure of the investigated samples are shown in Fig. 6.

Electron transparent PV specimens for *in-situ* heating experiments were ultrasonically cut, polished to ~70 μm, and Ar^+^-ion milled at 5 keV (gradually reduced to 2 keV) and 5° from the substrate side using a Gatan precision ion polishing system (PIPS) cooled by liquid nitrogen. CS specimens were prepared by the traditional ‘sandwich’ approach, which includes sample cutting into pieces, mounting into a Ti grid, gluing with a high-temperature-stability glue (Gatan G-1 epoxy), and polishing to ~50 μm. Ar^+^-ion milling was done from both sides applying the same milling steps as for plan-view samples.

The *in-situ* heating experiments were performed in the double-corrected Linköping FEI Titan^3^ 60–300 operated at 300 kV, by using a furnace type double tilt heating holder (Gatan Model 652). The PV sample *in-situ* heating started by pre-heating the sample at 500 °C for 10 min, then continuously heating at 600 °C for 3 h. The CS sample were pre-heated at 500 °C for 10 min, then continuously heating at 750 °C for 3 h and an additional at 900 °C for 1 h. In this case, a higher temperature (750 °C for CS while 600 °C for PV) was used in order to induce the decomposition reaction at similar speed as for the PV sample. The need for temperature adjustment with respect to CS sample is caused by the diverse TEM geometries (PV vs CS) and differences in thermal conductivities resulting in different actual temperature on the sample^25^.

The sample decomposition process was followed in real time by STEM - high angle annular dark field (STEM-HAADF) imaging as well as monochromated valence electron energy loss spectroscopy (VEELS) spectrum imaging (SI) using an energy spread of the primary electron beam of 0.2 eV. STEM-HAADF imaging was performed using 0.1 nA beam current which ensures tolerable electron dose and preserves the samples’ structure while imaging^6^. STEM–HAADF images were acquired using strong mass-contrast (Z-contrast) conditions, enhancing contrast and assonated peak intensities of FFT reflection from InAlN layer due to the larger scattering cross-section of the In atoms^26^. During *in-situ* heating, a series of overview and high-resolution (HR)STEM-HAADF images were recorded from CS samples at representative time intervals: 0 h, 0.5 h, 1 h, and 4 h, while for PV: 0 h, 1 h, 2 h, and 3 h.

The composition of the cross-sectional samples was monitored by STEM-VEELS SI and E_p_ mapping of before and after the full heating cycle. E_p_ follows an approximately linear dependence with respect to the alloys’ compositional as observed experimentally In_x_Al_1−x_N^17^ and calibrated according to calibrated according^19^, although to fully account for dependence, the usage of bowing parameter was suggested^27^,^28^. VEELS SIs were recorded using a probe convergence semi-angle of 20 mrad, which delivered sub-Ångström resolution with 0.3 nA current, 0.025 eV/channel energy dispersion, a collection semi-angle of 12 mrad and 0.005 s dwell time for each pixel. STEM-VEELS could not be applied to PV sample due to relatively small signal originating from the thin and embedded In_0.72_Al_0.28_N layer in comparison to the stronger and superimposed signals from the AlN buffer and capping layers (present above/below in the PV geometry).

The spatially resolved map of E_p_ was obtained from the VEELS SIs by an initial zero loss peak fitting and re-alignment of the spectrum image for energy drift, followed by Fourier-log deconvolution for plural scattering removal. Finally, applying a single 2 eV full width at half maximum (FWHM) Gaussian to the spectrum, the EELS spectrum was fitted by a nonlinear least-square (NLLS) curve-fitting method centered around the most intense part of the plasmon peak for extracting bulk plasmon peak energy with a fitting accuracy of 0.01 eV^29^.

## Additional Information

**How to cite this article:** Palisaitis, J. *et al*. Direct observation of spinodal decomposition phenomena in InAlN alloys during *in-situ* STEM heating. *Sci. Rep.* **7**, 44390; doi: 10.1038/srep44390 (2017).

**Publisher's note:** Springer Nature remains neutral with regard to jurisdictional claims in published maps and institutional affiliations.

## Supplementary Material

Supplementary Information
