# Supplementary material for: Direct observation of spinodal decomposition phenomena in InAlN alloys during in-situ STEM heating
Source: Sci Rep. 2017 Mar 14;7:44390. doi: 10.1038/srep44390 (PMC5349532; doi:10.1038/srep44390)
Supplement: Supplementary Information [file srep44390-s1.pdf]

Supplementary information for

Direct observation of spinodal decomposition phenomena in InAlN alloys during *in-situ* STEM heating

J. Palisaitis, C.-L. Hsiao, L. Hultman, J. Birch, and P.O.Å. Persson*

Thin Film Physics Division, Department of Physics, Chemistry and Biology (IFM), Linköping University, SE-581 83 Linköping, Sweden

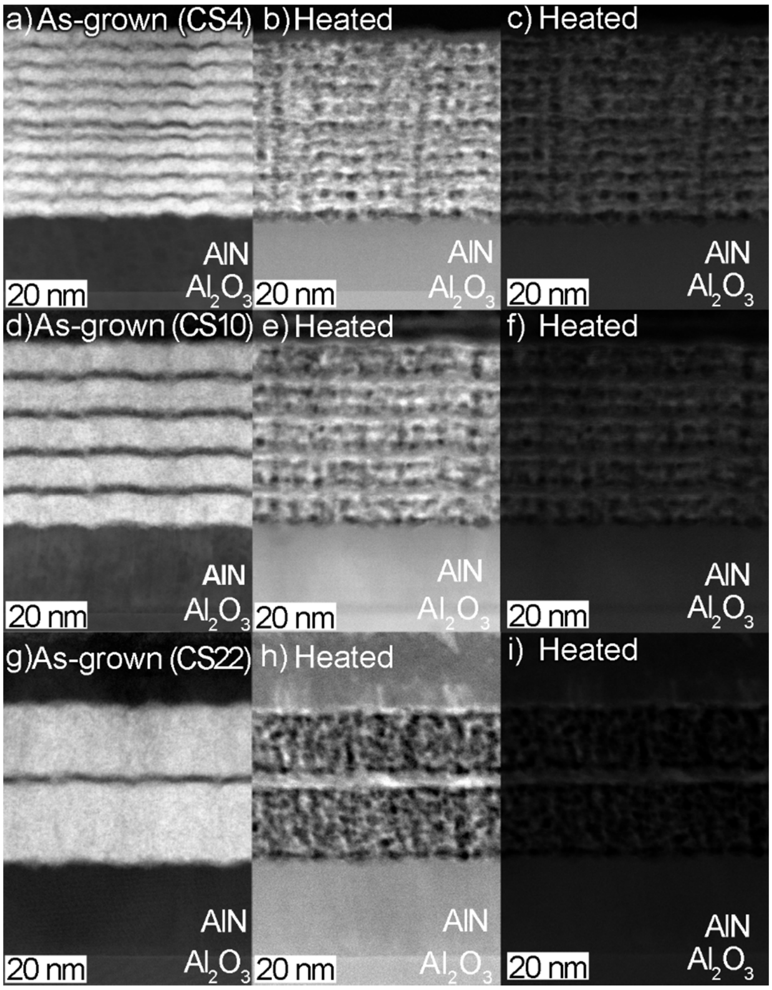

Fig. S1. Cross-sectional STEM-HAADF images of as-grown samples containing $In_{0.72}Al_{0.28}N$/AlN superlattices with different periods (a, d and g). The corresponding fully heated samples with enhanced STEM-HAADF contrast are shown in b, e and h, while with original STEM-HAADF image contrast are shown in b, e and h, respectively.

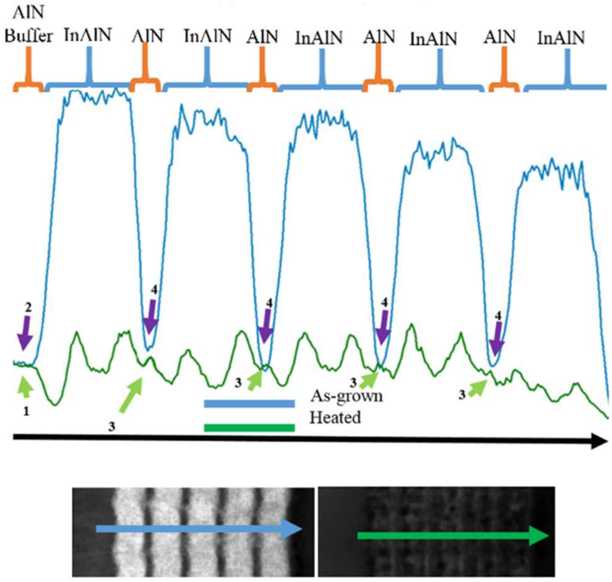

Fig. S2. Line intensity profiles obtained from STEM-HAADF images of CS10 samples before and after heating experiment. Thick AlN buffer layer can be regarded as references and are on the same intensity level for both as-grown (arrow #1) and heated (arrow #2) cases. Image intensity level coming from AlN interlayer showed good match in both cases (arrows #3 and #4) as well.
